# A Systematic Review of Clinical Validated and Potential miRNA Markers Related to the Efficacy of Fluoropyrimidine Drugs

**DOI:** 10.1155/2022/1360954

**Published:** 2022-08-23

**Authors:** Xiaomeng Sun, Jiani Chen, Xintao Chen, Qianmin Gao, Wei Chen, Xun Zou, Feng Zhang, Shouhong Gao, Shi Qiu, Xiaoqiang Yue, Houshan Yao, Xuan Liu, Mingming Li

**Affiliations:** ^1^Institutes of Biomedical Sciences, Fudan University, No. 131, Dongan Rd., Shanghai 200032, China; ^2^Department of Pharmacy, Changzheng Hospital, Naval Medical University, Shanghai 200003, China; ^3^Department of General Surgery, Changzheng Hospital, Naval Medical University, Shanghai 200003, China; ^4^Traditional Chinese Medicine Resource and Technology Center, Shanghai University of Traditional Chinese Medicine, Shanghai 201203, China; ^5^Department of TCM, Changzheng Hospital, Naval Medical University, Shanghai 200003, China

## Abstract

Colorectal cancer (CRC) is becoming increasingly prevalent worldwide. Fluoropyrimidine drugs are the primary chemotherapy regimens in routine clinical practice of CRC. However, the survival rate of patients on fluoropyrimidine-based chemotherapy varies significantly among individuals. Biomarkers of fluoropyrimidine drugs'' efficacy are needed to implement personalized medicine. This review summarized fluoropyrimidine drug-related microRNA (miRNA) by affecting metabolic enzymes or showing the relevance of drug efficacy. We first outlined 42 miRNAs that may affect the metabolism of fluoropyrimidine drugs. Subsequently, we filtered another 41 miRNAs related to the efficacy of fluoropyrimidine drugs based on clinical trials. Bioinformatics analysis showed that most well-established miRNA biomarkers were significantly enriched in the cancer pathways instead of the fluoropyrimidine drug metabolism pathways. The result also suggests that the miRNAs screened from metastasis patients have a more critical role in cancer development than those from non-metastasis patients. There are five miRNAs shared between these two lists. The miR-21, miR-215, and miR-218 can suppress fluoropyrimidine drugs'' catabolism. The miR-326 and miR-328 can reduce the efflux of fluoropyrimidine drugs. These five miRNAs could jointly act by increasing intracellular levels of fluoropyrimidine drugs'' cytotoxic metabolites, leading to better chemotherapy responses. In conclusion, we demonstrated that the dynamic changes in the transcriptional regulation via miRNAs might play significant roles in the efficacy and toxicity of the fluoropyrimidine drug. The reported miRNA biomarkers would help evaluate the efficacy of fluoropyrimidine drug-based chemotherapy and improve the prognosis of colorectal cancer patients.

## 1. Introduction

Colorectal cancer (CRC) is the third most commonly diagnosed malignancy and the second leading cause of cancer death worldwide [1]. The global burden of CRC is expected to increase to more than 2.5 million new cases in 2035 [1]. The incidence of CRC in developed countries, such as in Europe and North America, has stabilized and declined. In contrast, the incidence of CRC in developing countries is still on the rise [2], especially in China [3]. It is estimated that 4.3 million new cancer cases and 2.9 million new cancer deaths occurred in China in 2018 [2], meaning 30% and 40% higher cancer incidence and mortality than in the UK and USA [2]. Fluoropyrimidine-based (5-fluorouracil/5-FU, Capecitabine, Tegafur) drugs are the most commonly used chemotherapeutic agents in CRC treatment. They significantly improved the survival rates of CRC patients [4, 5]. However, many CRC patients will still experience recurrences or develop advanced diseases. The mutations in coding genes [6] and microRNAs (miRNAs) [7] were related to individual heterogenesis of drug efficacy and toxicity. Personalized therapy has been proved to aid clinicians in improving the treatment outcome [6]. The essential requirement is reliable marker systems with solid clinical evidence or precise molecular mechanisms.

The enzyme thymidylate synthase (TS) can methylate deoxyuridine monophosphate (dUMP) to form deoxythymidine monophosphate (dTMP). 5-FU acts as an inhibitor of TS by reducing the dTMP formation and ultimately blocking the formation of thymidine, an essential nucleoside for DNA replication and repair. Administration of 5-FU can rapidly induce cancer cell death via lack of thymidine [8]. The pharmacokinetics or pharmacodynamics of fluoropyrimidine drugs and their reactive metabolites are closely related to their efficacy [9]. These are controlled by enzymes and compounds that participate in drug transportation and metabolism. For instance, the calcium folinate can enhance 5-FU's cytotoxicity by providing exogenous folinate, stabilizing the 5-FU-TS complex [10].

Much effort has been made to screen for biomarkers that can affect or measure the activity of these effectors to predict chemotherapy response. MiRNAs can act as gene regulators that affect the tumor cell life cycle, such as growth, differentiation, and apoptosis. MiRNAs are small noncoding RNAs containing 21-24 nucleotides that play essential post-transcriptional regulatory roles in diverse organisms, including humans [11]. They affect protein function by combining with semi-complementary target mRNAs, resulting in mRNA destabilization and translation repression [12]. After the discovery of miRNA in the early-90s of twentieth century, the roles of miRNA in the pharmacology of standard chemotherapy drugs such as 5-FU were studied. Lorena Rossi and colleagues published one of the earliest studies in 2007 [13]. They proved that 5-FU could alter miRNA expression in malignant cells profoundly. These miRNAs include two of the most important miRNA markers, miR-21 and miR-200b, whose functions have been validated in many following observational studies since then. In the second decade of the twenty-first century, huge progress has been made to screen miRNA makers related to the efficacy or safety of 5-FU and 5-FU-based drugs. And a significant portion of these works was completed by European researchers such as Torben Frøstrup Hansen, who linked another important miRNA biomarker, miR126, with 5-FU's efficacy from a randomized phase III study with A total of 230 patients [14]. However, most therapy-associated miRNA biomarkers are deduced from molecular biological mechanisms based on cellular models, which leaves them lacking clinical validation. Besides, a single biomarker also lacks sensitivity and specificity. A miRNA panel could help increase the predictive efficiency [15].

Chen Lab is one of the pioneers in utilizing computational algorithms to discover small molecule drug-miRNA associations in batches. Dual-network collaborative matrix factorization (DCMF) [16], Ensemble of kernel ridge regression-based small molecule-miRNA association prediction (EKRRSMMA) [17], and bounded nuclear norm regularization for small molecule-miRNA associations prediction (BNNRSMMA) [18] methods were developed to fulfill the need, contributing to the miRNA panel discovery and validation efforts. Based on these sophisticated and subtle studies, to acquire a promising miRNA biomarker panel for predicting the efficacy of fluoropyrimidine drugs, each potential miRNA biomarker should have a clear biological function or should have been validated by retrospective or observational clinical trials. In this study, we reviewed miRNAs that may affect fluoropyrimidine drugs' metabolism and miRNAs that are associated with the response to fluoropyrimidine therapy. Five miRNAs meet both criteria, and their biological roles are further discussed.

## 2. Materials and Methods

### 2.1. Searching Strategy

We searched research articles on enzymes affected by miRNAs in the fluoropyrimidine drug metabolic pathway by the following strategy. Online databases including Embase, PubMed, Google Schooler, and Science Direct (updated to Sep 1, 2019) were searched [19]. We explicitly designed the searching strategy with the following terms: (“TS” OR “thymidylate synthase”) AND (“miRNA” OR “microRNA” OR “miR-” OR “microRNAs” OR “miRNAs”).  Table 1 shows a complete list of search keywords used in this article. Each result was recorded with title, date of publication, author list, and abstract for finner screening as described below.

Furthermore, we searched clinical trial outcomes related to the efficacy of fluoropyrimidine drugs according to the established paradigm of the Cochrane framework [20]. Online databases including Embase, PubMed, Google Scholar, and Science Direct (updated to Sep 1, 2019) were searched. A predefined searching strategy that outlined and combined the following terms was designed for this review: (a) in the title: (“colorectal” OR “colon” OR “rectum”) AND (“cancer” OR “tumor” OR “tumour” OR “Carcinoma” OR “neoplasia”) AND (“miRNA” OR “microRNA” OR “miR-” OR “microRNAs” OR “miRNAs”). Relevant reviews or articles on the citation list were independently screened to ensure completeness. Duplicate studies were combined before proceeding to the next round of filtering. b) Considering the most common validation for studies focusing on finding miRNAs to predict better chemotherapy outcomes is whether the potential marker could produce a good ROC curve with reasonable sensitivity and specificity based on regression analysis. We used the following keywords in Mendeley software to locate the publications with detailed efficiency assessments: (“survival” OR “response” OR “os” OR “PFS” OR “side” OR “adverse” OR “toxic” OR “effectiveness” OR “prognosis” OR “diagnosis” OR “diagnostic value” OR “detection” OR “biomarker” OR “sensitivity AND specificity” OR “ROC curve”) AND (“chemotherapy” OR “5-fu” OR “capecitabine” OR “fluoropyrimidine” OR “fluorouracil” OR “FOLFOX” OR “XELOX”) AND (patients). We summarized the search keywords and their representing categories in Table 1.

### 2.2. Filtering Strategy

The initial searched studies were further filtered based on the following criteria: **(**a) clinical research should base on humans; (b) all included patients should have had chemotherapy with fluoropyrimidine drugs; and (c) differences in survival should be related to miRNA expressions. Besides, the following studies were discarded: (a) duplicate publication; (b) case reports, letters to the editor, or review articles; and (c) studies with unqualified or insufficient clinical data.

**Figure 1:**
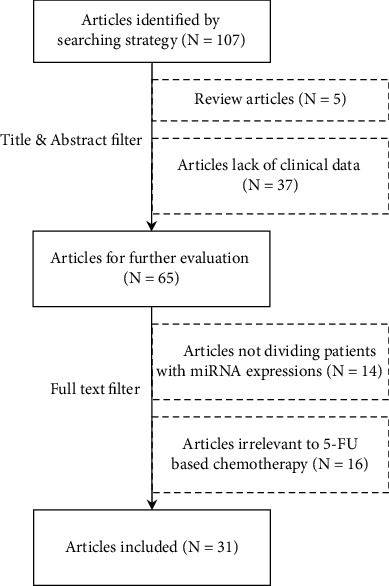
The literature searching and filtering workflow.

The flow chart of the entire literature filtering process is shown in Figure 1. The initial search returned 107 articles, of which five review articles were excluded. After carefully reviewing their titles and abstracts, 37 unrelated articles were excluded due to the lack of clinical data. The remaining 65 articles were available for further full-text manual check. Another 14 articles were excluded because the survival difference was unrelated to any miRNA expression. And patients from 16 articles did not receive chemotherapy with fluoropyrimidine drugs. In the end, there were 31 articles included in this review.

In the final 31 selected articles, candidate miRNAs and their corresponding target genes were summarized and enriched to the Kyoto Encyclopedia of Genes and Genomes (KEGG) pathways and Gene Ontology (GO) terms. The functional enrichment analysis was performed by the Database for Annotation, Visualization, and Integrated Discovery (DAVID) online tool [21, 22]. A number of matched genes larger than five and *P* values less than 0.05 (corrected by the two-side Bonferroni test) were considered significant.

## 3. Results

### 3.1. miRNAs Affecting the Metabolism of 5-FU

**Figure 2:**
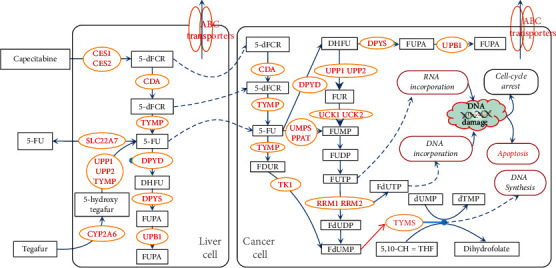
Metabolic pathway map of 5-FU. Abbreviations: 5-FU: 5-Fluorouracil; CES1: recombinant carboxylesterase 1; UPP1: uridine phosphorylase 1; 5′-dFCR: 5′-deoxy-5-fluorocytidine; CDA: cytidine deaminase; TYMP: thymidine phosphorylase; DPYS: dihydropyrimidinase; FUPA: 5-fluorouracil-hydantoic-acid; UPB1: recombinant beta-ureidopropionase; TK1: thymidine kinase 1; UMPS: uridine monophosphate synthetase; PPAT: phosphoribosyl pyrophosphate amido transferase; DHFU: dihydrofluorouracil; FUR: fluorouridine; FUMP: fluorouridine monophosphate; FUDP: fluorouridine diphospho; FUTP: fluorouridine triphosphate; FdUDP: fluorodeoxyuridine diphospho; FdUMP: fluorodeoxyuridine monophosphate; FdUTP: fluorodeoxyuridine triphosphate; dUMP: deoxy-uridine monophosphate; and dTMP: deoxy-thymidine monophosphate.

Based on the toxicity-related polymorphisms studies of fluoropyrimidines drugs proposed earlier [23–28], a metabolic pathway of 5-FU was drawn (Figure 2). We then searched for miRNAs that affect these metabolizing enzymes.

The cytotoxic metabolite of fluoropyrimidine drugs is fluorodeoxyuridine monophosphate (FdUMP), inhibiting the TS. 5-FU can be converted to FdUMP through different metabolic pathways (Figure 2). Thymidine phosphorylase (TP, or TYMP) controls one of the main metabolic pathways by converting 5-FU to fluorine de-oxidation pyridine (FUDR). FUDR can subsequently be converted to FdUMP catalyzed by thymidine kinase (TK). Another catalytic pathway converts the 5-FU to FdUMP through a more extended route, with a rate-limiting enzyme DPYD. The primary intermediate metabolites are fluorouridine diphosphate (FUDP) and fluorodeoxyuridine diphosphate (FdUDP), which could be further converted to fluorouridine triphosphate (FUTP) and fluorodeoxyuridine triphosphate (FdUTP). They can incorporate themselves into the newly formed DNA or RNA and suppress the normal replication and repair process. A previous study also showed that FUTP and FdUTP also contributed to the efficacy of fluoropyrimidine drugs [29].

We targeted 42 miRNAs that may affect the expression of enzymes within the fluoropyrimidine drug metabolic pathway (Table 2).

### 3.2. miRNAs Affecting the Efficiency of 5-FU

For the fluoropyrimidine drug efficacy-related miRNAs from the selected 31 articles, miRNAs with solid evidence from clinical trials were included. The relationship between chemotherapy response and miRNA expression level was confirmed by Kaplan-Meier plot with log-rank test or Cox regression *P* values less than 0.05. We filtered 41 miRNAs (Table 3) with necessary clinical information such as tumor stage and miRNA expression data. Eleven miRNAs were summarized from the studies in Asia. The median number of patients is 84. Six studies derived their conclusion from plasma samples, 24 from tissue, and one from both.

The miRNA related to fluoropyrimidine drug efficacy were listed in two subgroups based on CRC stages. MiRNAs in the first group were screened from patients without metastasis CRC (I, II, or III). MiRNAs in the second group were screened from patients with metastasis CRC (IV). If available, we recorded the number of patients within each stage (I, II, III, or IV) in the bracket. We marked these miRNAs according to the types of tissue (AMT: adjacent mucosal tissues; LR: local recurrences; metastases; plasma; and PT: primary tumors). Each miRNA expression level was tagged by its relationship with adverse clinical outcomes (shorter PFS, OS, or DFS). The statistical significance of each miRNA was annotated as superscript: for log-rank test, ∗, <0.05; ∗∗, <0.01; ∗∗∗, <0.001; and for COX test, #, <0.05; ##, <0.01; ###, <0.001. The origin of these studies was listed as the two-letter codes for countries and regions: AT, Austria; CN, China; DK, DENMARK; CZ, Czech; ES, Spain; FR, France; DE, Germany; JP, Japan; HK, Hong Kong; NL, Netherlands, NO, Norway; ES, Spain; NL, Netherlands; and PL, Poland.

The top 20 KEGG pathways based on fluoropyrimidine-efficacy-related miRNAs from the two subgroups (with metastasis and without metastasis) are listed in Table 4. Both enrichment results contain pathways directly related to the disease (including types of cancers). Many pathways regulate the normal cellular process, the abnormality of which might be the susceptible factor for cancer development. For miRNAs from the group without metastasis CRC, besides disease or cancer pathways, the FoxO signaling pathway, MAPK signaling pathway, autophagy, and PI3K-Akt signaling pathway are enriched, and their rank is within the top ten of all the enriched pathways. On the other hand, disease (especially cancer) pathways contribute the most to the miRNAs in the metastasis CRC cohort. The ranks of the FoxO signaling pathway and the MAPK signaling pathway were reduced from 2 to 4 and 4 to 17, respectively.

In addition, those fluoropyrimidine-efficacy-related miRNAs from the two subgroups were also subjected to GO term enrichment (Table 5). Based on the results, miRNAs from the two cohorts showed similar cellular component (CC) and molecular functions (MF) characteristics. For CC, they both have intracellular organelle, membrane-bounded organelle, and intracellular membrane-bounded organelle. For MF, they both have enzyme binding, regulatory region nucleic acid binding, and transcription regulatory region sequence-specific DNA binding. However, they showed quite a different biological process (BP). MiRNAs from the cohort without metastasis patients are mainly enriched in metabolic-related functions, such as regulating the cellular metabolic process, the primary metabolic process, and the nitrogen compound metabolic process. On the other hand, miRNA from the cohort with metastasis patients is mainly enriched in cell cycle control mechanisms, such as G1/S transition of mitotic cell cycle, mitotic cell cycle, cell morphogenesis, and cell morphogenesis involved in differentiation.

## 4. Discussion

This study first summarized a list of 42 miRNAs that may affect fluoropyrimidine drug metabolism based on literature research. Subsequently, we have created another list of 41 miRNAs related to fluoropyrimidine drugs' efficacy based on clinical trials according to the Cochrane framework. By comparing the two sets, we found that miR-21, miR-215, miR-218, miR-326, and miR-328 could affect the metabolic pathways of 5-FU and their expressions were associated with CRC survival after fluoropyrimidine adjuvant chemotherapy.

MiR21 is a marker for better efficacy of fluoropyrimidine drugs for CRC patients with and without metastasis. It can suppress the expression of dihydropyrimidine dehydrogenase (DYPD) and thymidine phosphorylase (TP) [30]. DPYD is a crucial enzyme in fluoropyrimidine drugs metabolism [31], which takes charge of the detoxifying process of 5-FU in the liver. A low DPD level can increase internal exposure to 5-FU and its cytotoxicity, resulting in better efficacy. On the other hand, Capecitabine is almost wholly absorbed in the gastrointestinal tract, metabolized to 5-deoxy 5-cytosine nucleoside (5'-DFCR), and finally converted into 5-FU by thymidine phosphorylase (TP) [32, 33]. A low TP level may reduce the catabolism of fluoropyrimidine drug, resulting in extended exposure of 5-FU and its cytotoxic intermediate metabolites. Furthermore, several pieces of literature from Czech [34], German [35], Japanese [35], and Chinese [36] have confirmed that the increased expression of miR-21 was significantly correlated with good outcomes of adjuvant chemotherapy. Thus, miR-21 is a solid marker for using fluoropyrimidine drugs after CRC surgery.

For miR-215 and miR-218, they are positively related to better chemotherapy response in patients without metastasis. In fluoropyrimidine drug metabolism, they suppress the expression of thymidylate synthetase (TS) [37, 38]. TS is an enzyme that catalyzes the conversion of deoxyuridine monophosphate (dUMP) to deoxythymidine monophosphate (dTMP) (Figure 2). The dTMP is the fundamental building material for DNA and RNA synthesis. Suppressed TS expression can cause the cells to be more sensitive to genotoxic stress, further activating programmed cell death pathways, resulting in DNA fragmentation [39]. MiR-215 and miR218 could make the tumor cells more sensitive to chemotherapy. Three clinical trials from different regions have indicated that induced expression of miR-215 and miR-218 could lead to a good curative effect and survival [40–42].

For miR-326 and miR-328, their high plasma expressions are positively related to good chemotherapy response in patients with/without metastasis [43]. In fluoropyrimidine drug metabolism, miR-326 and miR-328 can suppress ATP-binding cassette (ABC) subfamily C member 1 (ABCC1) and ATP-binding cassette (ABC) subfamily G member 2(ABCG2), subsequently. This may lead to the increased intracellular concentration of fluoropyrimidine drugs and their metabolites. And the induced cytotoxicity increases as well (Figure 2). Since the clinical evidence was concluded based on plasma samples, miR-326 and miR-328 may cause an overall suppressed efflux of fluoropyrimidine drugs and their metabolites.

Of the 42 miRNAs that may affect fluoropyrimidine drug metabolism, 37 lack direct clinical evidence on their predictive effect on efficacy of fluoropyrimidine drugs. Detection limits and other experimental factors might limit the discovery of their potential prediction effects. They could be screened as potential biomarkers by future properly designed clinical experiments.

For the 41 miRNAs related to fluoropyrimidine drugs' efficacy based on clinical trials, 36 of them may not affect fluoropyrimidine drug metabolism enzymes. This result suggests that proteins other than those from the fluoropyrimidine drug metabolism pathway may also contribute equally or even more to the efficacy of fluoropyrimidine drugs. Consistent with this finding, we have found that several urine endogenous metabolites can predict fluoropyrimidine drugs' adverse effects [44]. These adverse effects, such as hand-foot syndrome, are predictors of better chemotherapy response alone [45, 46]. Based on the KEGG pathway and GO term enrichment results, miRNAs screened from the patients with and without metastasis showed similar results. These two subgroups of miRNA enrich both the FoxO signaling pathway and the MAPK signaling pathway. The abnormality of these pathways is a susceptible factor for cancer development. The difference is that the latter subgroup of miRNAs enriched more disease or cancer pathways, which may result from the advanced stage of the tumor.

In the future, the studies on miRNA biomarkers could be improved in the following aspects. We think what comes first is that more biological mechanism experiments are needed to reveal the actual function of miRNA markers in cancer development or drug pharmacology. Since a number of the miRNA markers were only derived from clinical screening studies, they could be either the actual markers or merely the outcome of cancer development or drug metabolism. This question is complicated because one miRNA may affect many genes, and several miRNAs may regulate one gene. A review by Xing Chen may provide valuable guidance for future research on this aspect. This review summarized and discussed not just the databases of the experimentally validated or potential small molecule-miRNA associations but also four experimental techniques used in the past few years to search for small-molecule inhibitors of miRNAs [47]. Secondly, with the rapid advances in omics techniques, future clinical screening studies could be designed for multi-omics biomarkers, including miRNA, since other biomolecules such as DNA, proteins, and metabolite also contribute to the final phenotype. Last but not least, screening miRNAs from normal tissues other than tumors may provide more informative clues. Accumulating evidence suggests that the efficacy and safety of chemotherapy are not solely dependent on drug metabolism but also on the overall physiological functions [44, 48–53]. Individual differences in cell maintenance, proliferation, and immune function also influence the response to chemotherapy.

## 5. Conclusions

In conclusion, we have found that 41 miRNAs are related to fluoropyrimidine drugs' efficacy with solid clinical evidence. They are promising candidate markers for predicting fluoropyrimidine drugs' efficacy in the future clinical application of personalized medicine. The miRNAs screened from metastasis CRC patients have a more critical role in cancer development based on bioinformatic analysis than those screened from non-metastasis CRC patients. Among the 41 miRNAs, miR-21, miR-215, and miR-218 can suppress fluoropyrimidine drugs' catabolism; miR-326 and miR-328 can reduce the efflux of fluoropyrimidine drugs. Together, these five miRNAs can increase the intracellular levels of cytotoxic metabolites of fluoropyrimidine drugs, leading to better chemotherapy response.

## Figures and Tables

**Table 1:** Keywords used in the literature searching strategy.

Type of keywords	Keywords used in literature searching
Target enzyme-related	TS
	Thymidylate synthase

Treatment-related	Chemotherapy
	5-fu
	Capecitabine
	Fluoropyrimidine
	Fluorouracil
	FOLFOX
	XELOX
	Patients

Phenotype-related	Colorectal
	Colon
	Rectum
	Cancer
	Tumor
	Tumour
	Carcinoma
	Neoplasia

MicroRNA-related	miRNA
	microRNA
	miR-
	microRNAs
	miRNAs

Validation-related	Survival
	Response
	Os
	PFS
	Side
	Adverse
	Toxic
	Effectiveness
	Prognosis
	Diagnosis
	Diagnostic value
	Detection
	Biomarker
	Sensitivity
	Specificity
	ROC curve

**Table 2:** The list of miRNAs affecting the expression of 5-FU metabolic enzymes.

Affected protein	MicroRNA	Related cancer (cell lines or patients)
ABCC5(+)	miR-101 [74]	HCC
ABCC1(+)	miR-199 [74]	HCC
ABCC1(-)	miR-326 [75]	Breast cancer
ABCC2(-)	miR-397 [76]	Hepatoblastoma cell
ABCC3(-), ABCC6(-)	miR-9 [77]	Glioma cell
ABCC4(-)	miR-125 [74]	Hepatocellular carcinoma
ABCG2(-)	miR-212 [78]	Myelogenous leukemia
ABCG2(-)	miR-328 [78–81]	Breast cancer, retinoblastoma, myelogenous leukemia, and CRC
ABCG2(-)	miR-519 [79, 82, 83]	Colon cancer, breast cancer, and retinoblastoma
DPYD(-)	miR-134 [84]	HCC, lung cancer
DPYD(-)	miR-494 [85]	Colon cancer
DPYD(-)	miR-582 [84]	HCC
DPYD(-), P-gp(-)	miR-302 [86, 87]	HCC and breast cancer
P-gp(-)	miR-103 [88]	Gastric cancer
P-gp(-)	miR-107 [88]	Gastric cancer
P-gp(-)	miR-129 [89]	Gastric cancer
P-gp(+)	miR-130 [90]	Ovarian cancer
P-gp(-)	miR-137 [91]	Breast cancer
P-gp(-)	miR-138 [92]	Leukemia
P-gp(-)	miR-298 [93]	Breast cancer
P-gp(-)	miR-30 [94]	Gastric cancer
P-gp(-)	miR-331 [95]	Chronic myelogenous leukemia
P-gp(-), ABCB1(-)	miR-451[96–98]	Breast cancer, CRC
P-gp(-)	miR-506 [99]	CRC
P-gp(-), ABCG2(-)	miR-145 [100, 101]	Colon carcinoma
P-gp(-), ABCG2(-), ABCG5(-)	miR-200 [102, 103]	Breast cancer and melanomas
TP(-), DPYD(-)	miR-21 [30]	CRC
TS(-)	miR-192 [104]	CRC
TS(-)	miR-196 [37]	Rectal cancer
TS(-)	miR-197 [105]	CRC
TS(-)	miR-203 [106]	CRC
TS(-)	miR-215 [37, 104, 107–110]	CRC, soft tissue sarcoma, renal cancer, and head and neck cancer
P-gp(+)	miR-218 [38]	CRC
TS(-)	miR-24 [107]	Soft tissue sarcoma
TS(-)	miR-433 [111]	HCC
TS(-)	miR-450 [37]	Rectal cancer
TS(-)	miR-99 [37]	Rectal cancer
ABCC5(-)TS(-)	Let-7e [37, 74]	Rectal cancer, HCC
TS(-), ABCC3(-)	miR-192 [104, 112]	CRC and esophageal adenocarcinoma
TS(-), ABCC3(-)	miR-193 [112]	Esophageal adenocarcinoma
TS(-), ABCC3(-)	miR-378 [112]	Esophageal adenocarcinoma
TS(-), ABCG2(-)	miR-520 [80, 113–115]	HCC, pancreatic cancer, and retinoblastoma
TS(-), DPYD(-), P-gp(-), ABCC3(-)	miR-27 [37, 38, 74, 93, 95, 96, 102, 105, 116, 117]	CRC, HCC, lung cancer, gastric cancer, breast cancer, esophageal adenocarcinoma, leukemia, and ovarian cancer

The effect of miRNAs on each enzyme's expression was noted as “+” for inducing and “-” for suppressing. Abbreviations: CRC: colorectal cancer; HCC: hepatocellular carcinoma.

**Table 3:** The list of miRNAs relating to the efficacy of 5-FU.

miRNA	*N*	Region	Sources (N)	Survival	Expression	Stage (*N*)
*Without metastasis*						
miR-1300 [54]	85	PL	PT	DMFS^#^	—	I-II
miR-939 [54]	85	PL	PT	DMFS^#^	—	I-II
miR-135b [54]	85	PL	PT	DMFS^##^	+	I-II
miR-1296 [54]	85	PL	PT	DMFS^##^	+	I-II
miR-539 [54]	85	PL	PT	DMFS^##^	+	I-II
miR-572 [54]	85	PL	PT	DMFS^##^	—	I-II
miR-21 [35]	145	DE	PT	OS^#^	+	II
miR-215 [40]	71	ES	PT	DFS^∗∗^^,##^	+	II
miR-103a-3p [40]	71	ES	PT	DFS^∗^^,#^	+	II
miR-103a-3p [40]	71	ES	PT	DFS^#^	+	II
miR-143-5p [40]	71	ES	PT	DFS^#^	+	II
miR-103a-3p [40]	71	ES	PT	DFS^#^	+	II
miR-143-5p [40]	71	ES	PT	DFS^#^	+	II
miR-143-5p [40]	71	ES	PT	DFS^∗^^,#^	+	II
miR-21 [36]	125	CN	PT	DFS^∗∗∗^	+	II-III
miR-21 [35]	87	JP	PT	OS^#^	+	II-III
miR-218 [40]	63	CN	PT	PFS^∗∗^/OS^∗∗∗^	—	II-III
miR-17-5p [55]	240	CN	PT	OS^##^	+	II-III
miR-320e [56]	167	ES	PT	OS^##^/DFS^##^	+	II-III
miR-625-3p [57]	77	DK	PT	OS^∗^	+	II-III
miR-148a [58]	201	ES	PT	DFS^#^	—	II-III
miR-148a [58]	201	ES	PT	DFS^#^	—	II-III
miR-141 [59]	56	ES	Plasma	DFS^∗^/OS^∗^	—	I-II (35), III(15)
miR-200c [59]	56	ES	Plasma	DFS^∗^/OS^∗^	—	I-II (35), III(15)
miR-342-3p [60]	322	CN	Plasma	DFS^###^/OS^##^	+	I-III
miR-652-3p [60]	322	CN	Plasma	DF ^###^/OS^##^	+	I-III
miR-501-3p [60]	322	CN	Plasma	DFS^###^/OS^##^	+	I-III
miR-328-3p [60]	322	CN	Plasma	DFS^###^/OS^##^	+	I-III
miR-4772-3p [61]	84	US	Plasma	OS^#^	—	II-III
*With metastasis*						
miR-126 [62]	83	DK	PT	OS^∗∗^/PFS^∗∗∗^	+	I-III (3), IV(86)
miR-199b [63]	60	CN	PT	OS^∗^^,#^	—	I-IV
miR-17-5p [64]	81	CN	PT	OS^∗∗∗^^,#^	+	I-IV
miR-143 [65]	52	AT	PT	PFS^∗^	—	II-IV
miR-21 [66]	32	JP	PT	PFS^∗^	+	IV
miR-31-3p [67]	45	FR	PT	PFS^∗^	+	IV
miR-107 [68]	78	ES	PT	PFS^#^	+	IV
miR-889 [68]	78	ES	PT	PFS^#^/OS^##^	—	IV
miR-337-5p [68]	78	ES	PT	PFS^##^	+	IV
miR-148a [58]	71	ES	PT	OS^∗^^,#^	—	IV
miR-99a-3p [68]	78	ES	PT	PFS^#^	+	IV
miR-31 [69]	221	CN	PT, AMT	DFS^∗^/OS^∗^	+	II-IV
miR-365 [70]	76	CN	PT, AMT	DFS^∗^	—	I-IV
miR-133a [71]	125	HK	PT, AMT	OS^∗^	+	I-IV
miR-20a-5p [72]	88	NZ	PT (80), LR (3), metastases (5)	PFS^#^	+	I-IV
miR-92a-3p [72]	88	NZ	PT (80), LR (3), metastases (5)	PFS^#^	+	I-IV
miR-92b-3p [72]	88	NZ	PT (80), LR (3), metastases (5)	PFS^#^	+	I-IV
miR-30a-5p [72]	88	NZ	PT (80), LR (3), metastases (5)	PFS^#^	+	I-IV
miR-98-5p [72]	88	NZ	PT (80), LR (3), metastases (5)	PFS^#^	+	I-IV
miR-17-5p [72]	88	NZ	PT (80), LR (3), metastases (5)	PFS^#^	+	I-IV
miR-126 [73]	68	DK	Plasma	PFS^∗^	+	IV
miR-148 [43]	150	NO	Plasma	PFS^##^	+	IV
miR-326 [43]	150	NO	Plasma	PFS^##^/OS^##^	+	IV
miR-27b [43]	150	NO	Plasma	PFS^##^	+	IV

**Table 4:** Top 20 significant KEGG pathways enriched by fluoropyrimidine drug efficacy–related miRNAs in CRC cohorts with/without metastasis.

Without metastasis				With metastasis			
KEGG pathway	Genes	Ratio (%)	*P* value	KEGG pathway	Genes	Ratio (%)	*P* value
MicroRNAs in cancer	62	20.00	9.87E-11	Chronic myeloid leukemia	22	28.95	6.36E-09
FoxO signaling pathway	36	27.48	7.13E-10	Cellular senescence	30	18.75	2.46E-07
Cellular senescence	40	25.00	1.21E-09	TGF-beta signaling pathway	22	23.40	5.48E-07
MAPK signaling pathway	57	19.39	3.38E-09	FoxO signaling pathway	26	19.85	8.85E-07
Autophagy	33	24.09	2.47E-07	Non-small cell lung cancer	18	27.27	1.09E-06
Proteoglycans in cancer	42	20.49	3.02E-07	MicroRNAs in cancer	43	13.87	1.31E-06
PI3K-Akt signaling pathway	60	16.95	3.1E-07	Pancreatic cancer	19	25.00	1.99E-06
Hepatitis B	36	22.22	4.58E-07	Signaling pathways regulating pluripotency of stem cells	26	18.18	5.82E-06
AGE-RAGE signaling pathway in diabetic complications	26	26.00	2.53E-06	Pathways in cancer	59	11.11	1.22E-05
Pancreatic cancer	22	28.95	3.94E-06	Proteoglycans in cancer	31	15.12	2.4E-05
Pathways in cancer	76	14.31	5.99E-06	Hepatocellular carcinoma	27	16.07	4.4E-05
Colorectal cancer	23	26.74	9.64E-06	Glioma	17	22.67	5.26E-05
Kaposi sarcoma-associated herpesvirus infection	37	19.58	9.82E-06	Hepatitis B	26	16.05	7.44E-05
Glioma	21	28.00	1.52E-05	Cell cycle	22	17.74	9.85E-05
Human cytomegalovirus infection	41	18.22	1.78E-05	Prostate cancer	19	19.59	0.000119
Chronic myeloid leukemia	21	27.63	1.94E-05	MAPK signaling pathway	37	12.59	0.000177
Bladder cancer	15	36.59	2.18E-05	Gastric cancer	24	16.11	0.000187
Prostate cancer	24	24.74	2.41E-05	AGE-RAGE signaling pathway in diabetic complications	19	19.00	0.00019

Genes related to miRNA from the two CRC cohorts were subjected to KEGG pathway enrichment analysis. The top 20 pathways with gene numbers higher than five and *P* values less than 0.05 (corrected by the two-side Bonferroni test) were listed here.

**Table 5:** Top five significant gene ontology (GO) terms enriched by fluoropyrimidine drug efficacy related miRNAs in CRC cohorts with/without metastasis.

	Without metastasis				With metastasis			
	GO term	Genes	Ratio (%)	*P* value	GO term	Genes	Ratio (%)	*P* value
CC	Intracellular organelle	791	6.01	2.60E-23	Intracellular organelle	1079	8.20	2.15E-29
CC	Nucleus	534	6.88	5.02E-21	Membrane-bounded organelle	1059	8.20	1.55E-27
CC	Membrane-bounded organelle	772	5.98	2.24E-20	Intracellular membrane-bounded organelle	959	8.43	2.23E-25
CC	Intracellular membrane-bounded organelle	705	6.20	2.78E-20	Nucleoplasm	444	10.63	4.40E-23
CC	Nucleoplasm	333	7.97	4.07E-19	Nuclear lumen	505	10.16	7.21E-23
MF	Enzyme binding	219	9.23	5.89E-18	Enzyme binding	278	11.72	6.49E-18
MF	Regulatory region nucleic acid binding	113	10.82	2.79E-12	Phosphotransferase activity, alcohol group as acceptor	192	13.41	2.22E-17
MF	Sequence-specific DNA binding	136	9.79	8.82E-12	Transferase activity, transferring phosphorus-containing groups	218	12.20	3.41E-15
MF	Transcription regulatory region sequence-specific DNA binding	112	10.74	9.23E-12	Regulatory region nucleic acid binding	148	14.18	6.59E-15
MF	Sequence-specific double-stranded DNA binding	114	10.45	2.42E-11	Transcription regulatory region sequence-specific DNA binding	147	14.09	1.88E-14
BP	Regulation of cellular metabolic process	510	7.77	6.99E-34	G1/S transition of mitotic cell cycle	52	19.48	9.10E-09
B.P.	Regulation of primary metabolic process	484	7.62	1.38E-28	Negative regulation of transcription by RNA polymerase II	132	14.12	7.85E-13
B.P.	Regulation of nitrogen compound metabolic process	473	7.68	1.55E-28	Mitotic cell cycle	146	13.21	7.20E-12
B.P.	Regulation of metabolic process	532	7.29	4.96E-28	Cell morphogenesis	135	11.93	1.70E-07
B.P.	Regulation of macromolecule metabolic process	496	7.38	4.72E-26	Cell morphogenesis involved in differentiation	96	11.82	1.77E-04

Genes related to miRNA from the two CRC cohorts were subjected to G.O. enrichment analysis. The top G.O. terms with gene numbers higher than five and *P* values less than 0.05 (corrected by the two-side Bonferroni test) were listed here. Three ontology sources were analyzed in this step: cellular component (CC), molecular function (M.F.), and biological process (B.P.).

## Abbreviations

5-FU: 5-fluorouracil
CRC: Colorectal cancer
DAVID: Visualization and iIntegrated dDiscovery
dTMP: Deoxythymidine monophosphate
dUMP: Deoxyuridine monophosphate
DYPD: Dihydropyrimidine dehydrogenase
FdUMP: Fluorodeoxyuridine monophosphate
GO: Gene oOntology
KEGG: Kyoto eEncyclopedia of gGenes and gGenomes
miRNA: microRNAMicroRNA
TP: Thymidine phosphorylase
TS: Thymidylate synthase.
